# Madin‐Darby canine kidney cell sialic acid receptor modulation induced by culture medium conditions: Implications for the isolation of influenza A virus

**DOI:** 10.1111/irv.12671

**Published:** 2019-08-07

**Authors:** Sarah W. Nelson, Joshua N. Lorbach, Jacqueline M. Nolting, Jason W. Stull, Daral J. Jackwood, Ian C. Davis, Andrew S. Bowman

**Affiliations:** ^1^ Veterinary Preventive Medicine The Ohio State University Columbus Ohio; ^2^ Food Animal Health Research Program, The Ohio State University Wooster Ohio; ^3^ Veterinary Biosciences, The Ohio State University Columbus Ohio

**Keywords:** influenza, MDCK cells, serum‐free medium, sialic acid receptors

## Abstract

**Background:**

The influenza A virus (IAV) binds to α‐2,3‐ and α‐2,6‐linked sialic acid (SA) receptors expressed by Madin‐Darby canine kidney (MDCK) cells. The receptor distribution may therefore be important in regulating IAV propagation. Serum‐free medium (SFM) avoids variability in conventional culture medium containing fetal bovine serum (FBS), which can have variable composition and may contain endotoxins. However, little is known about the distribution of SA receptors on cells maintained in SFM.

**Objectives:**

We assessed the influence of culture media on MDCK cell SA receptor distribution along with the effect of SA receptor distribution on IAV recovery. We hypothesized that SFM would increase the proportion of α‐2,6‐linked SA receptors present and alter isolate recovery.

**Methods:**

Madin‐Darby canine kidney cells were cultured in medium containing FBS and two SFMs. Cell surface distribution of α‐2,6‐ and α‐2,3‐linked receptors was determined using flow cytometry. Recovery of swine‐ and avian‐lineage IAVs from MDCK cells maintained in each medium was quantified as TCID_50_.

**Results:**

Madin‐Darby canine kidney cells cultured in UltraMDCK SFM expressed both SA receptors and supported the growth of both swine‐ and avian‐lineage IAVs. Cells maintained in other medium inconsistently expressed each receptor and the avian IAV grew to lower titers in cells cultured with FBS.

**Conclusions:**

Medium conditions altered the distribution of SA receptors present on MDCK cells and affected IAV recovery. Culture in UltraMDCK SFM resulted in cells expressing both receptors and IAVs grew to higher titers than in the other culture condition, indicating that this medium may be useful for culturing IAV from multiple species.

## 1 INTRODUCTION

Influenza A virus (IAV) is a common pathogen that infects numerous species and adversely affects both public and animal health. Thousands of people become infected with seasonally circulating IAVs each year resulting in substantial morbidity and mortality.^1^ Outbreaks of influenza in commercial poultry and swine operations result in large economic losses and can facilitate reassortment events leading to zoonotic transmission with pandemic potential.^2^, ^3^ Continued surveillance and characterization of IAV from animal host species are needed to moderate the risk to public health.

Galactose at the receptor binding site of IAV hemagglutinin (HA) protein binds to α‐2,3‐linked or α‐2,6‐linked sialic acid (SA) on host cells to facilitate infection.^4^ The type of SA receptor linkages found on host cells contributes to a transmission barrier for IAV strains between host species.^5^ Avian‐lineage IAVs generally have preferential binding to SA cellular receptors in the α‐2,3‐linked conformation, while swine‐ and human‐lineage IAVs bind SA receptors in the α‐2,6‐linked conformation.^6^ Therefore, changes in the type of linkage of SA (ie, α‐2,3‐linked vs α‐2,6‐linked) and the level of expression of each type of SA on the cell surface could ultimately impact the efficiency of IAV infection and replication.

Several techniques have been developed to isolate IAVs including the use of embryonated chicken eggs and immortalized cell lines.^7^, ^8^, ^9^, ^10^, ^11^, ^12^, ^13^ Madin‐Darby canine kidney (MDCK) cell lines are widely available, easily amplify in culture, and are commonly used for isolating IAVs from a variety of species.^12^, ^14^ Previous studies indicated that MDCK cells cultured in the presence of fetal bovine serum (FBS) express both α‐2,6‐linked and α‐2,3‐linked SA receptor types and showed that some cells co‐express both receptors.^9^, ^14^ In contrast, another study found that 98% of MDCK cells adapted to serum‐free media (SFM) expressed only α‐2,6‐linked SA receptors, with 2% of cells expressing both α‐2,3‐ and α‐2,6‐linked SA receptors.^15^ However, detailed characterization of SFM‐adapted MDCK cells is needed because variations in SFM preparations may cause the cells to express different amounts of each SA receptor, altering IAV culture success. Indeed, MDCK SIAT1 cells, which overexpress α‐2,6‐linked SA receptors, improve isolation rates for human IAV versus standard MDCK cells.^16^ Increasing the recovery of IAV from culture systems is important for the timely detection of seasonal and novel IAV to prevent further spread and permit appropriate medical treatment for symptomatic individuals. We hypothesized that culture in SFM would increase the proportion of MDCK cells expressing α‐2,6‐linked SAs and subsequently increase the recovery of mammalian origin IAV. We assessed MDCK cell SA receptor distributions over serial passages in commercially available SFM using flow cytometric analysis and quantified IAV recovery.

## 2 MATERIALS AND METHODS

### 2.1 Monitoring sialic acid receptor expression on MDCK cells

Madin‐Darby canine kidney cells (Sigma‐Aldrich, St. Louis, MO, USA; cat. no. 84121903‐1VL) were expanded in medium containing FBS (Gibco; cat. no. 10082‐139) as previously described.^15^ This medium (medium A) consisted of minimum essential medium (MEM) with Earle's balanced salts (EBSS), l‐glutamine supplemented with 1× sodium pyruvate, 1× non‐essential amino acids, and 10% heat‐inactivated FBS. To prevent the growth of *Mycoplasma,* the culture medium was supplemented with MycoZap prophylactic (Lonza Walkersville, Inc, Walkersville, MD, USA) during the first four passages after removal of cells from cryopreservation. MDCK cells were recovered from culture flasks by treating with 0.25% trypsin and 0.1% EDTA. After cells detached from the flask, trypsin was inhibited with the addition of medium A.

**Figure 1:**
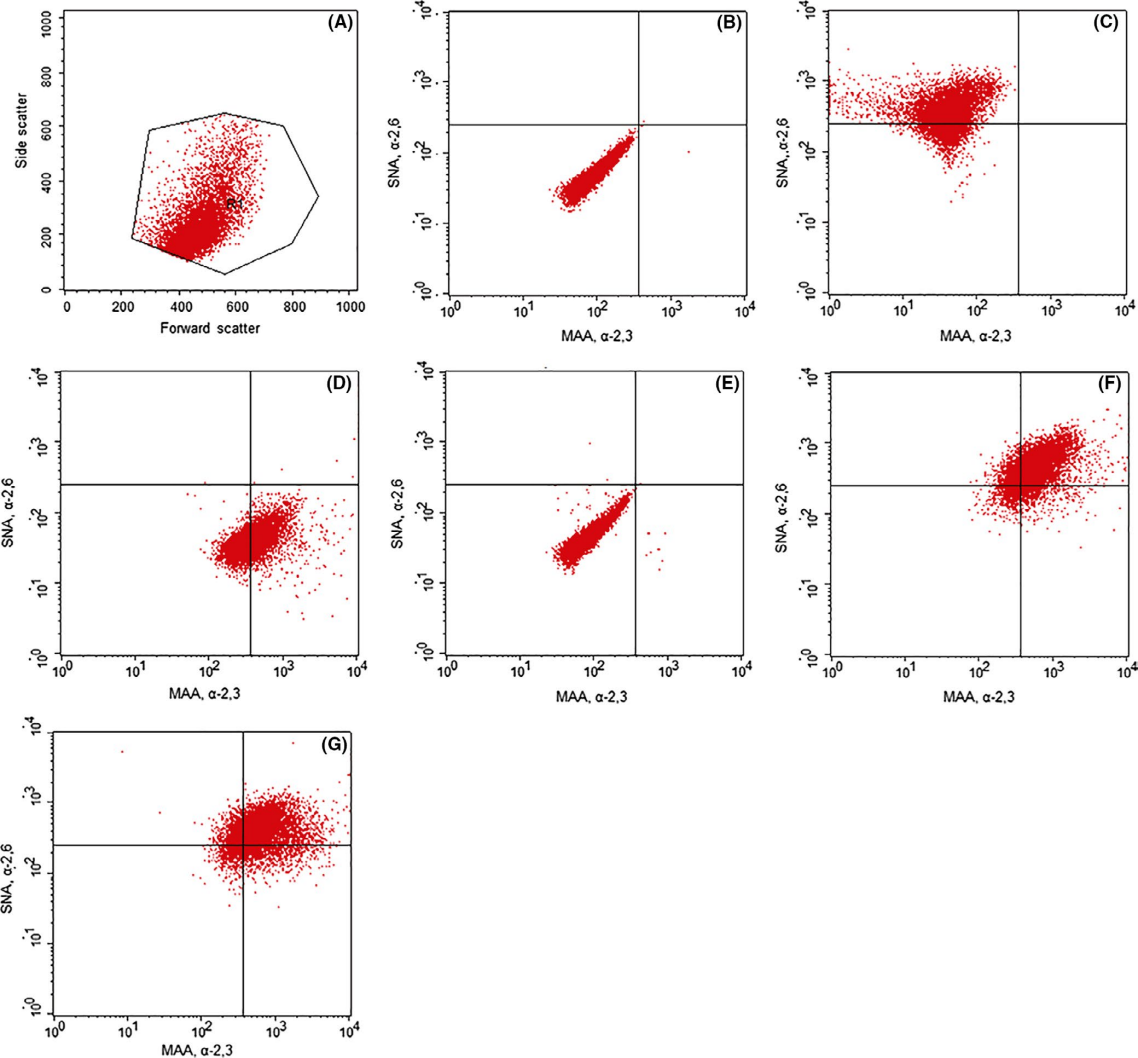
The flow cytometric controls and analysis of MDCK cells. [Panel A] The forward scatter versus side scatter gate shows the size and granularity of the cell population. Small debris and large doublets were excluded from analysis. The same gate was used for all samples. [Panel B] An unstained negative control sample. [Panel C] A sample single stained with biotinylated *Sambucus nigra* (SNA) and streptavidin‐phycoerythrin control, which binds to α‐2,6‐linked sialic acids. FL2‐ is detecting phycoerythrin (PE) or the SNA stain. [Panel D] A sample single stained with *Maackia amurensis* (MAA) and streptavidin‐phycoerythrin, which binds to α‐2,3‐linked sialic acids. FL‐1 is detecting fluorescein isothiocyanate (FITC) or MAA stain. [Panel E] A sample streptavidin‐phycoerythrin negative control. [Panel F] MDCK cells maintained in medium A dual stained with biotinylated SNA lectin, MAA lectin, and streptavidin‐phycoerythrin. [Panel 1G] MDCK cells maintained in medium B dual stained with biotinylated SNA lectin, MAA lectin, and streptavidin‐phycoerythrin

After expansion, cells were distributed into three medium groups: A, B, and C. While one‐third of the cells remained in medium A, equal portions were transitioned to medium B (UltraMDCK™ SFM, Lonza Walkersville, Inc, Walkersville, MD, USA) and medium C (OptiPRO™ SFM, Life Technologies, Carlsbad, CA, USA) as previously described.^15^, ^17^, ^18^ MDCK cells were cultured in triplicate in each of the three mediums. Cells were passed twice weekly with cell concentrations sufficient to make flasks confluent at either 3 or 4 days. The percentage of MDCK cells expressing α‐2,6‐linked and α‐2,3‐linked SA receptors from passages 4 through 25 was measured by flow cytometry using the BD FACSCalibur as described.^15^ On each passage day immediately after detaching the cells from the flask and inhibiting trypsin with medium A, trypan blue was used to count cells and determine cell viability. 1 × 10^6^ cells per sample were washed with PBS containing 5% FBS and 0.02% sodium azide (PBS/azide). The cells were incubated with biotinylated *Sambucus nigra* (SNA) lectin (10 µg/mL, cat. no. B‐1305) from Vector Laboratories (Burlingame, CA, USA) and/or fluorescein isothiocyanate (FITC)‐conjugated *Maackia amurensis* (MAA) lectin (100 µg/mL, cat. no. F‐7801‐2) from EY Laboratories (San Mateo, CA, USA). The SNA lectin is specific for α‐2,6‐linked SA on the cell surface, while the MAA lectin is specific for α‐2,3‐linked SA. PBS/azide was added to the unstained control and the streptavidin‐phycoerythrin only control. Samples were incubated with the stains at 4°C in the dark for 30 minutes and then washed with PBS/azide. Wash was removed, and streptavidin‐phycoerythrin (100 µg/mL, cat. no. F0040) from R&D Systems (Minneapolis, MN, USA) was added to all samples and controls except the unstained control. The streptavidin‐phycoerythrin conjugate allows detection of biotinylated SNA. Samples and controls were mixed by vortexing and incubated in the dark for 30 minutes at 4°C, then centrifuged and washed with 300 µL PBS/azide. Wash was removed, and cytofix (250 µL, cat. no. 554655) from BD Biosciences (San Jose, CA, USA) was added to all samples and controls. Cells were fixed for 30 minutes at 4°C in the dark, then washed, resuspended, and stored in the dark in 500 µL PBS/azide until flow cytometric analysis could be performed. When 7‐amino‐actinomycin D (cat. no. 00‐6993 Thermo Scientific, Waltham, MA, USA) was used for live/dead cell discrimination, it was added between the staining and fixation steps{Fetterhoff TJ, 1993 #3873}. This discrimination was performed using 100 µL PBS/Azide, which was added to the cell pellet with 5 µL 7‐amino‐actinomycin D, mixed and incubated in the dark for 30 minutes at 4°C, then washed and fixed as above. The number of events captured was 5 × 10^4^. The gate was drawn to include the majority of live, single cells and exclude large doublets and small debris (Figure 1D). The same gate was used for all samples. The neuraminidase *Arthrobacter ureafaciens* from Millipore (Burlington, MA, USA cat. no. 480716‐250MIU) was used prior to staining to remove sialic acids from the cells to determine staining specificity. Cells were washed and suspended in 500 µL PBS/azide with 0.5 µL neuraminidase for 30 minutes at 37°C, then washed and stained. The FACSCalibur compensation settings were kept the same for all samples. Logistic regression was used to compare the percentage of cells expressing both receptors across passages five through 25. For medium A, the percentage of cells expressing both receptors after 3 days in culture was compared to the percentage of cells expressing both receptors after 4 days in culture across passages five through 25. These percentages were also compared to the percentage of cells expressing both receptors when cultured in medium B, across passages five through 25. The percentages for media A and B were compared to the percentage of cells expressing both receptors in medium C, regardless of passage day, across passages 5 through 25. The geometric mean fluorescent intensity of each channel was averaged to compare the staining intensity and investigate the expression level of each SA receptor for cells maintained in each medium for each passage day.

### 2.2 Effect of culture media on sialic acid receptor expression

To determine whether alterations in SA receptor distribution are a consequence of depletion of medium nutrients due to cell growth, MDCK cell SA receptor distribution during culture in medium A and B was evaluated. MDCK cells were seeded at densities that ensured confluence at 1 day (high‐8 × 10^6^ cells/T25 flask), 3 days (medium high‐1.5 × 10^6^ cells/T25 flask), 4 days (medium‐9.25 × 10^5^ cells/T25 flask), or 7 days (low‐1 × 10^5^ cells/T25 flask) post‐seeding (Figure S1). Forty‐five individual flasks for each medium were seeded at different densities to be confluent after 1 day (three individual flasks), 3 days (nine individual flasks), 4 days (12 individual flasks), or 7 days (21 individual flasks). Flasks from each medium and concentration were evaluated by flow cytometry each day for 7 days. Flasks that were not confluent until day three were evaluated by flow cytometry on days one and two before reaching confluency. Flasks were not monitored after becoming confluent. Medium C was not included due to the increased variability in SA receptor expression seen in experiment 1 when compared to our other SFM, medium B. Protein concentrations of aliquots of supernatant were determined using the Pierce bicinchoninic acid (BCA) protein assay from Thermo Scientific (Waltham, MA, USA, cat. no. 23227). The supernatant protein concentration from the flasks was compared to the protein concentration of the stock medium using logistic regression in Stata for medium A and B separately.

### 2.3 Effect of receptor distributions on infectivity

MDCK cells were cultured in medium A and B separately as described above in a third experiment. Cells in T25 flasks were stained to determine the distributions of α‐2,6‐linked and α‐2,3‐linked SA receptors upon confluency 1 day after flask seeding. Trypsin is an important component of IAV viral growth medium because it is needed to cleave the HA protein allowing the virus to infect other cells. Because FBS inhibits trypsin activity, it was not included in the viral growth medium of cells cultured in medium A for this experiment. Eight different IAVs were inoculated into media A and B: A/swine/Ohio/12TOSU447/2012(H3N2), A/green‐winged teal/Ohio/175/1986(H2N1), A/American black duck/Ohio/16OS0658/2016(H7N3), A/mallard duck/Ohio/16OS0672/2016(H8N4), A/common goldeneye/Wisconsin/16OS4147/2016(H10N3), A/green‐winged teal/Mississippi/16OS5996/2016(H5N2), A/mallard/Ohio/17OS1740/2017(H3N8), and A/American green‐winged teal/Ohio/17OS1850/2017(H4N6) as previously described.^15^ Cell monolayers were evaluated for cytopathic effects (CPE) by inverted light microscopy 72 hours post‐inoculation. TCID_50_/mL values were calculated using the Reed and Muench method.^19^ The above was repeated at the subsequent cell passage. TCID_50_/mL values across the combinations of medium type and IAV were compared using the Kruskal‐Wallis test, if significant pairwise Mann‐Whitney tests were used to compare between combinations. All statistical analyses were performed in Stata Version 14.0 (StataCorp, College Station, TX).

## 3 RESULTS

### 3.1 Monitoring sialic acid receptor expression on MDCK cells

#### 3.1.1 Medium A

**Figure 2:**
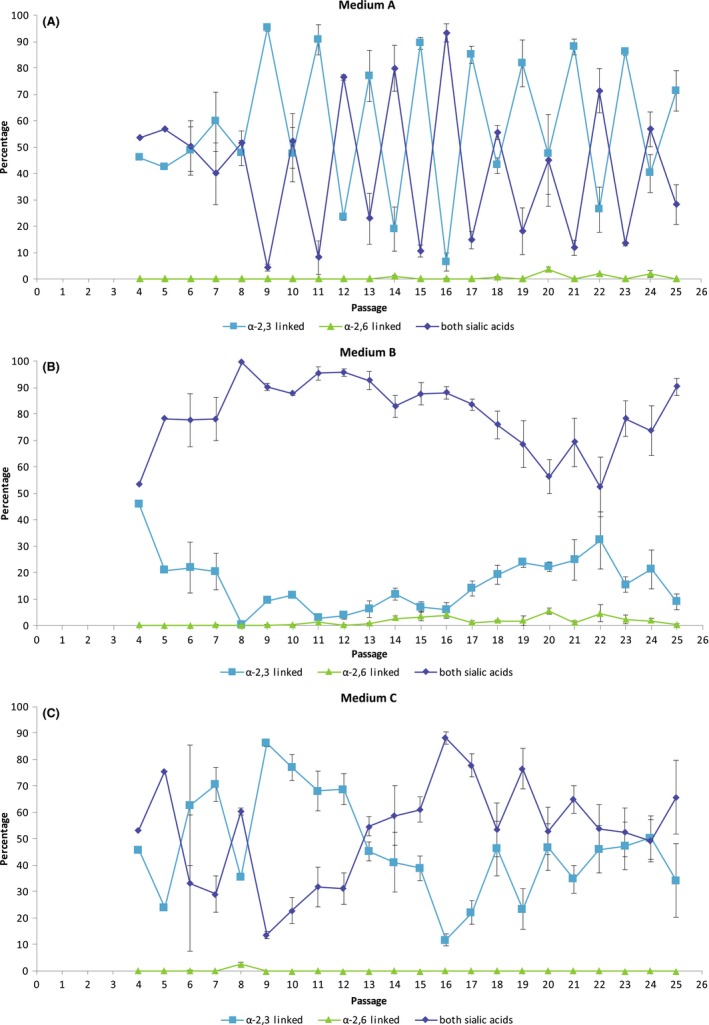
Flow cytometry was used to define the percentage of cells expressing only α‐2,3‐linked SA receptors, only α‐2,6‐linked SA receptors, or both SA receptors from passage 4 to passage 25. Cells were transitioned to each SFM during passages 4 thru 7. [Panel A] The percentage of MDCK cells expressing each SA or both when cultured in medium A. [Panel B] The percentage of MDCK cells expressing each SA or both when cultured in medium B. [Panel C] The percentage of MDCK cells expressing each SA or both when cultured in medium C. The error bars show the standard deviation of the mean of three flasks

**Figure 3:**
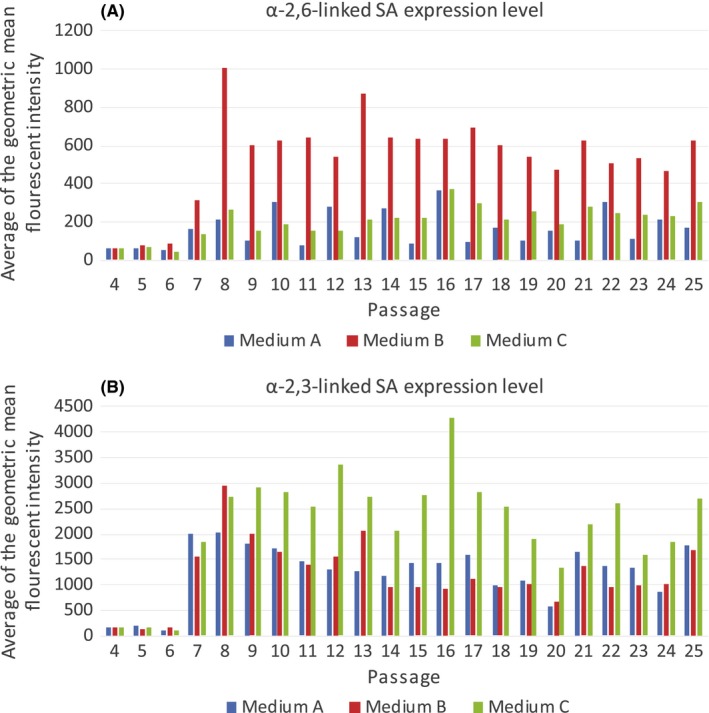
[Panel A]. Average of the geometric mean fluorescent intensity for the SNA stain, indicating the α‐2,6‐linked SA expression level for each medium A, B, and C. [Panel B] Average of the geometric mean fluorescent intensity for the MAA stain, indicating the α‐2,3‐linked SA expression level for each medium A, B, and C

Cells were passed twice weekly (Monday and Thursday) resulting in flow cytometric testing on an alternating three‐ or four‐day schedule. Cytometry controls are shown (Figure 1). Culture of MDCK cells in medium A resulted in highly variable proportions of cells expressing each SA receptor, which alternated between passages (Figure 2A). For passages with 4 days of growth, a mean of 63% (range 45% to 93%) of the cells expressed both receptors, 36% (range 6% to 49%) expressed only α‐2,3, and 0.9% (range 0% to 4%) expressed only α‐2,6‐linked SA receptors. Cells confluent after 3 days had a mean of 21% (range 4% to 57%) expressing both receptors, 79% (range 42% to 95%) only α‐2,3, and 0.006% (range 0% to 0.04%) only α‐2,6‐linked SA receptors. The percentage of cells expressing both receptors after 4 days in culture (mean 63%) was statistically different from the percentage of cells expressing both receptors after 3 days in culture (mean 21%) (*P* ≤ .005). MDCK cells maintained in medium A stained less intensely with the SNA stain than medium B cells, indicating fewer α‐2,6‐linked SA receptors per cell than medium B cells (Figure 3A). Medium A cells also stained less intensely with the MAA stain than medium C cells, indicating fewer α‐2,3‐linked SA receptors per cell than medium C cells (Figure 3B).

#### 3.1.2 Medium B

When MDCK cells were maintained in medium B, a mean of 80% (range 52% to 100%) expressed both receptors regardless of time since passage (Figure 2B), 16% (range 0.4% to 46%) only expressed α‐2,3‐linked, and 2% (range 0.02% to 6%) expressed only α‐2,6‐linked SA receptors. The percentage of cells expressing both receptors (mean 80%) was statistically different from the percentage of cells expressing both receptors cultured in medium A after three (mean 21%) or 4 days (mean 63%) in culture (*P* = .002, *P* ≤ .005, respectively). Cells maintained in medium B stained more intensely with the SNA stain than medium A and C cells, indicating the presence of more α‐2,6‐linked SA receptors per cell than cells maintained in medium A or C (Figure 3A) even though most cells expressed both SAs.

#### 3.1.3 Medium C

Culture in medium C resulted in variable SA receptor expression (Figure 2C), but unlike cells cultured in medium A, rapid passage dependent cycling was not observed. A mean of 52% (range 14% to 88%) of cells expressed both receptors, 47% (range 12% to 86%) expressed only α‐2,3‐linked SAs, and an average of 0.1% (range 0% to 3%) expressed only α‐2,6‐linked SA receptors. The percentage of cells expressing both receptors (mean 52%) was statistically different from the percentage of cells expressing both receptors cultured in medium A after 3 days (mean 21%) (*P* ≤ .005), medium A after 4 days (mean 63%) (*P* = .016), and medium B (mean 80%) (*P *= <.005). Cells maintained in medium C stained more intensely with the MAA stain than medium A and B cells, indicating that these cells have more α‐2,3‐linked SA per cell (Figure 3B).

### 3.2 Effect of culture media on sialic acid receptor expression

**Figure 4:**
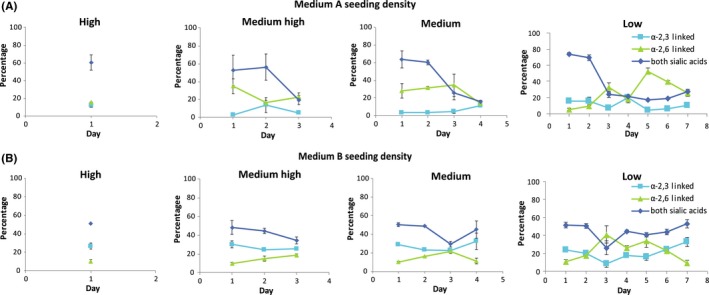
SA receptor expression on distributions of MDCK cells expressing α‐2,6‐linked, α‐2,3‐linked, or both SA receptors on cells maintained in medium containing FBS (medium A [Panel A]) and one serum‐free medium (medium B [Panel B]) when plated at different densities. [Panel A] The percentage of MDCK cells expressing each SA receptor or both when cultured in medium A and plated at different densities. [Panel B] The percentage of MDCK cells expressing each SA or both when cultured in medium B and plated at different densities. For the high‐density flasks, cells were confluent, trypsinized 24 h after seeding the flasks, and stained for flow cytometry. For the medium‐ to high‐density flasks, 3 flasks were trypsinized and stained 24 h post‐seeding, then another 3 flasks each day until no flasks remained. Cells were not confluent until 72 h post‐seeding. For the medium‐density flasks, 3 flasks were trypsinized and stained 24 h post‐seeding, then another 3 flasks each day until no flasks remained. Cells were not confluent until 96 h post‐seeding. The same was performed for the low‐density flasks. Cells were not confluent until 168 h post‐seeding. The error bars indicate the standard deviation around the mean of three flasks. Medium A‐maintained cells showed different percentages of cells expressing each SA receptor or both on different days. Medium B‐maintained cells consistently expressed both SA receptors

Based on the results from the first experiment, we sought to determine whether differences in MDCK cell SA receptor over time in the different media are a consequence of progressive nutrient depletion. In this experiment, cells cultured in medium A were seeded into 45 individual flasks so that flasks would be confluent after 1, 3, 4, or 7 days. Each day, SA receptor expression was evaluated in three flasks from each group by flow cytometry. Irrespective of initial seeding density, a high proportion of MDCK cells maintained in medium A expressed both receptors 1 day post‐seeding (Figure 4A). Interestingly, however, the proportion of cells co‐expressing both SA receptors declined after day 2 post‐seeding (at all densities). An average of 62% (range 52% to 74%) of cells expressed both receptors for the first 2 days after seeding, but this dropped to 21% (range 16% to 27%) on days three through seven.

The same method used above for medium A was applied to medium B. MDCK cells maintained in medium B expressed high proportions of both receptors regardless of initial seeding density (Figure 4B). 44% (range 26% to 53%) of cells cultured in medium B expressed both receptors, 18% (range 10% to 41%) expressed only α‐2,6‐linked SAs, and 24% (9% to 33%) expressed only α‐2,3‐linked SAs over the course of the experiment. In this experiment, some cells did not express either SA receptor. Culture in medium B resulted in more consistent MDCK cell SA expression profile distributions when compared to culture in medium A.

Culture supernatant protein concentrations were measured at the time of cell harvesting for flow cytometry to determine the extent of nutrient depletion resulting from prolonged cell culture. When MDCK cells were maintained in medium A, the supernatant protein concentration ranged from 3.8 to 4.2 mg/mL, with stock medium A testing at 3.9 mg/mL (Figure S2). The supernatant protein concentration of the medium B‐maintained flasks ranged from 0.6 to 0.8 mg/mL, while stock medium B tested at 0.8 mg/mL. When cells were maintained in medium B, the average supernatant protein concentration decreased slightly after 3 days in culture (0.7 mg/mL) compared to stock medium B (0.8 mg/mL), but this decrease was not statistically significant (*P* = .2).

### 3.3 Receptor distribution affects infectivity

**Figure 5:**
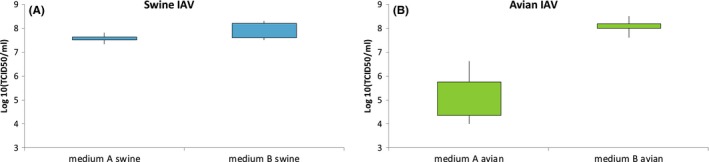
Replication of swine‐ and avian‐lineage IAVs in MDCK cells cultured in medium A and B. Data are shown as log‐transformed TCID_50_/mL. Each box represents 3 replicates from each trial. This figure combines the data from the two successive passages to illustrate the mean for each virus and medium combination

The swine‐lineage IAV grew to higher titers in medium B (mean 1 × 10^8^ TCID_50_/mL)‐maintained cells than cells cultured medium A (mean 3.8x10^7^ TCID_50_/mL) (Figure 5) (*P* = .068). All the avian‐lineage IAVs except for A/green‐winged teal/Ohio/175/1986(H2N1) grew to similar titers (within one log difference) in cells maintained in medium A and medium B (Figure S3). The exception, A/green‐winged teal/Ohio/175/1986(H2N1), grew to higher titers in medium B‐maintained cells (mean 1.4 × 10^8^ TCID_50_/mL) compared to growth in medium A (mean 9.5 × 10^5^ TCID_50_/mL) (*P* = .0049).

## 4 DISCUSSION

The use of SFM is increasingly popular to avoid the variability and contaminants associated with FBS.^8^ Transitioning cells in culture to SFM has the potential to alter the sensitivity of IAV recovery systems because changes in culture medium can alter the expression of SA receptors on cell membranes.^20^ Since IAVs from different species typically prefer binding to either α‐2,3‐linked or α‐2,6‐linked SA receptor types, cells that express both could be useful for the isolation of IAV from diverse species. In our study, culture in medium B resulted in cells commonly expressing both receptors, indicating that this brand of SFM might be useful for the culture of MDCK cells to isolate IAVs from diverse host species. Culture in medium B also increased the α‐2,6‐linked SA receptor expression per cell indicating that this medium might be particularly useful to increase the efficiency of isolation and recovery of mammalian origin IAVs. In contrast, variable receptor expression was observed when cells were cultured in medium A and medium C, making them less predictable and less suitable for IAV isolation, particularly at later time points post‐seeding: Our data indicate that cells cultured in medium A may only be effective if isolating IAV 1 or 2 days post‐seeding while cells cultured in medium B could be useful for up to 7 days post‐seeding. 7‐amino‐actinomycin D was used to verify that <4% of cells included in the gate for analysis were dead. Cells were treated with neuraminidase in an effort to determine staining specificity. We were able to remove a majority of SA from the cells with neuraminidase by reducing the percentage of cells staining from 91% to 31%, given that live MDCK cells continually produce new SA and the increase in dead cells following neuraminidase treatment likely resulted in 31% being an artificially high percentage of cells staining; therefore, we believe non‐specific staining in our study is low The supernatant protein concentrations were different between the media groups, but it remains unclear whether medium protein concentration influenced the distributions of α‐2,6‐linked and α‐2,3‐linked SA receptors on MDCK cells. The protein concentration did not significantly change during the trial, but the SA receptors expressed by cells cultured in medium A did fluctuate while SA receptors expressed by cells cultured in medium B did not. Overall, our findings indicate that MDCK cell SA receptor expression and IAV recovery efficiency may be significantly impacted by the composition of the culture medium. The implications of this are that different IAVs may or may not be recovered from MDCK cell culture systems depending on their receptor preference and the culture medium selected. IAV surveillance programs should be aware of the potential bias and variability imposed by culture medium.

It is important to note, especially from a diagnostic perspective, that the swine‐ and one avian‐lineage IAV grew to higher titers in medium B‐cultured cells than in medium A‐cultured cells. While most avian‐lineage IAVs preferentially bind to α‐2,3‐linked SA receptors, the A/green‐winged teal/Ohio/175/1986(H2N1) isolate used in the infectivity experiment might not bind efficiently to α‐2,3‐linked SA receptors that were present on the medium A‐maintained MDCK cells. This avian IAV previously grew to high titers in MDCK cells and poorly infected mice,^21^ which are known to express predominantly α‐2,3‐linked SA receptors in their lungs.^22^ Interestingly, A/green‐winged teal/Mississippi/16OS5996/2016(H5N2) has a substitution at position 226 in the HA protein that is supposed to increase binding to α‐2,6‐linked SA receptors.^23^ While not statistically significant, this isolate did grow better in medium B‐maintained cells (3.16 × 10^7^ TCID_50_/mL) compared to medium A‐maintained cells (7.13 × 10^6^ TCID_50_/mL). The other five avian‐lineage IAVs did not have this substitution, and the TCID_50_/mL values in medium A and B were all within half a log TCID_50_/mL. The binding preference of each IAV isolate used in these studies would have been valuable when interpreting the results. In future studies, receptor binding assays could be performed or previously characterized isolates could be used to help correlate IAV titer with SA distributions present on the cells. One group has proposed that glycan shape is more important for receptor binding than the angle or structure of the SA.^24^ Consequently, there are likely other binding receptors that were not investigated in this study, which may account for the results described herein. We were surprised to find that MDCK cells maintained in medium B mostly expressed α‐2,6‐linked SA receptors in the infectivity experiment. While different lots of MDCK cells were used for each of the experiments, we cannot explain this alteration. There are other SFMs that were not tested in this study that may perform better than medium B or medium C for the isolation of IAV. Additional experimentation is required to fully understand the extent to which culture media affects virus isolation.

Cell culture medium is an essential component of viral isolation systems as it contains all the nutrients cells require to grow, but formulations vary and deliver nutrients in different forms. Developed in 1959, the formula for minimum essential medium (MEM) or Eagle's growth medium contains all the components necessary to grow mammalian cell lines in laboratory culture systems.^25^ MEM is still commonly used today and may be supplemented with the specific amino acids, hormones, or metabolites that a particular cell line requires. FBS is commonly added because it contains many growth factors (eg, epidermal growth factor, fibroblast growth factor, nerve growth factor, endothelial cell growth factor, insulin‐like growth factors, transforming growth factors). However, the use of FBS can be problematic because it is an animal‐harvested product and each lot varies in composition and endotoxin contamination.^26^ We suspect that the cycling of receptor distributions seen in experiment 1, with cells cultured in medium A, was due to variability in the lot of FBS used in that medium. One hypothesis is that the cells in this experiment were using nutrients in medium A in a way that caused the cells to change receptor expression from one passage to the next. SA expression is dependent on nutrients available and processing by the Golgi.^27^ One group showed that cells cultured with modified SA derivatives will express modified SA after 48 hours.^20^ While additional experimentation is required to confirm this, our findings highlight the significant impact of changes in culture conditions on MDCK cell SA receptor profiles.

While we do not fully understand all the factors that affect the SA expression of MDCK cells, differences were seen when cells were cultured in medium A, B, or C in the SA linkage and level of expression. If a culture system does not provide the required SA receptors, IAV may not be recovered or may require multiple passages and adaptation of the virus for successful isolation. Our results demonstrate that cell culture medium used in a virus isolation system can affect the efficiency of an IAV surveillance program. Our data indicate that medium B may provide MDCK cells with an environment that allows them to consistently express both α‐2,3‐ and increased levels of α‐2,6‐linked SA receptors and successfully grow IAV from different species. Understanding which SA receptors are present on cells in the culture system used to isolate IAVs is important for the recovery of novel IAVs. It may be beneficial to verify SA receptor expression prior to using cells for the recovery of IAV.

## CONFLICT OF INTERESTS

The authors declare no conflicts of interest with respect to the research, authorship, and/or publication of this article.
