# Supplementary material for: Madin‐Darby canine kidney cell sialic acid receptor modulation induced by culture medium conditions: Implications for the isolation of influenza A virus
Source: Influenza Other Respir Viruses. 2019 Aug 7;13(6):593–602. doi: 10.1111/irv.12671 (PMC6800301; doi:10.1111/irv.12671)
Supplement: Supplementary file 1 [file IRV-13-593-s001.docx]

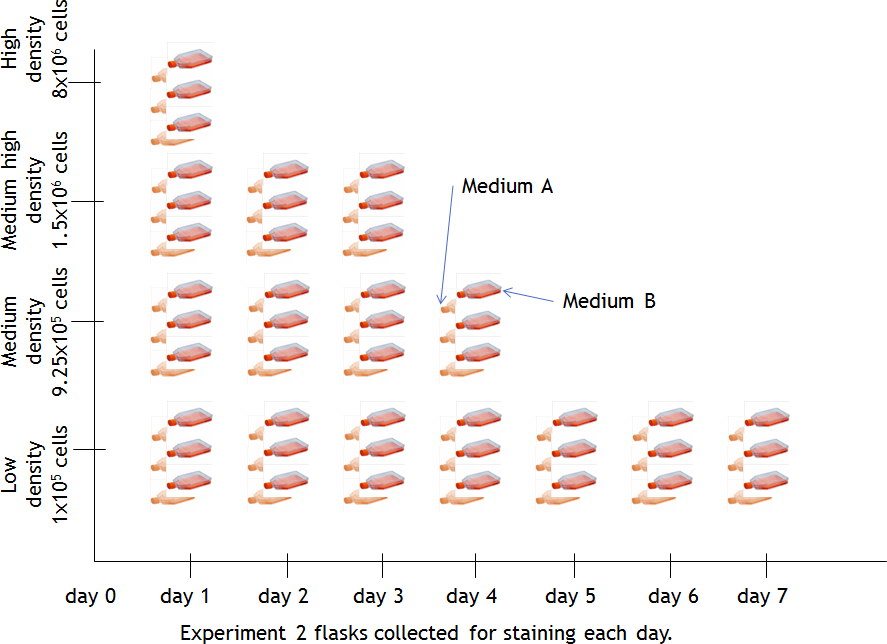


Supplemental Figure 1. Illustration of experiment 2 flasks collected each day for staining for flow cytometry. All flasks were seeded on day 0. High density flasks were confluent after 24 hours.

Medium high density flasks were not confluent until 3 days post seeding, but were stained and evaluated on days 1, 2, and 3. Medium density flasks were weren’t confluent until day 4, but were stained and evaluated on days 1, 2, 3, and 4. Low density flasks were not confluent until day 7, but were stained and evaluated on days 1 thru 7.


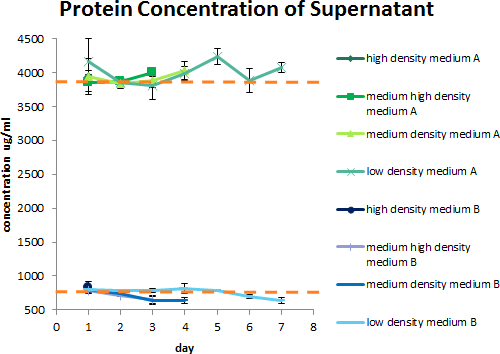


Supplemental Figure 2. Average supernatant protein concentrations of MDCK cells seeded at different densities maintained in media A or B. The error bars are the standard deviation about the mean of 3 flasks. The protein concentration of the stock media is shown with the dashed lines.

**Log 10(TCID50/ml)**

Supplemental Figure 3. Replication of avian-lineage IAVs in MDCK cells cultured in medium A and


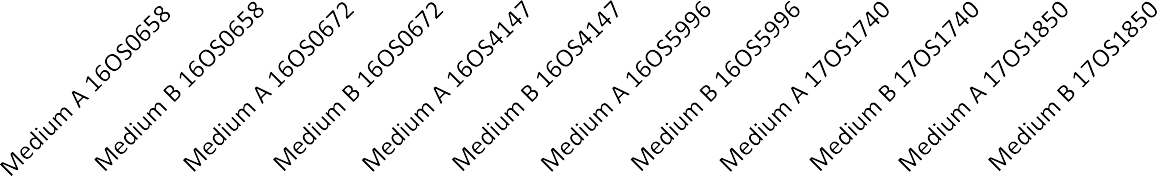


**Avian-lineage IAV replication**

9

8

7

6

5

4

3

**Medium and avian IAV combinations**

B. Data is shown as log-transformed TCID50/mL. Each box represents 3 replicates from each trial. This figure combines the data from the two successive passages to illustrate the mean for each virus and medium combination.
